# Influence of Reversed Fatigue Loading on Damage Evolution of Cross-Ply Carbon Fibre Composites

**DOI:** 10.3390/ma12071153

**Published:** 2019-04-09

**Authors:** Gordon Just, Ilja Koch, Martin Brod, Eelco Jansen, Maik Gude, Raimund Rolfes

**Affiliations:** 1 Institute of Lightweight Engineering and Polymer Technology (ILK), Technische Universität Dresden, Holbeinstr. 3, 01307 Dresden, Germany; ilja.koch@tu-dresden.de (I.K.); maik.gude@tu-dresden.de (M.G.); 2 Institute of Structural Analysis (ISD), Leibniz Universität Hannover, Appelstr. 9a, 30167 Hannover, Germany; m.brod@isd.uni-hannover.de (M.B.); e.jansen@isd.uni-hannover.de (E.J.); r.rolfes@isd.uni-hannover.de (R.R.)

**Keywords:** CFRP, composite, crack growth, delamination, failure, fatigue, fracture, load reversal, microcracking, residual stresses

## Abstract

Microcrack formation and delamination growth are the main damage mechanisms in the fatigue of composites. They lead to significant stiffness loss, introduce stress concentrations and can be the origin of subsequent damage events like buckling or fibre breakage, especially in case of shear and compression stresses during load reversal. Fatigue experiments of carbon fibre reinforced laminates were conducted at several stress ratios and analysed in terms of crack and delamination growth. These investigations were accompanied by microscopic imaging, digital image correlation and finite element modelling to take into account the effects of residual stresses and crack closure. It was found that residual stresses significantly change the local stress ratio in off-axis layers and lead to residual crack opening of inter fibre cracks. These cracks remain open and close under high compression loadings only. Furthermore, crack formation under pulsating compression loading turned out to be driven by residual stresses leading to perpendicular cracks as observed under pure tension loading. The experimental findings further confirm the severe detrimental effect of tension-compression loading on crack formation and delamination growth compared to pulsating tension-tension or compression-compression loads.

## 1. Introduction

High performance composite structures usually undergo complex loadings throughout their service lifetime. Whereas failure of composite materials under static loading conditions is fairly well understood and many damage models exist [1,2,3,4,5,6,7,8], the damaging process becomes more complex in case of fatigue loading. It is common sense, that the failure mechanisms inter fibre failure (further on termed as microcracks), delamination and fibre failure remain the same in fatigue, but their order of occurence and magnitude may vary between static and fatigue loading [9,10]. Hence, many models have been published, which can be distinguished by the required tests for calibration, modelling approach and model complexity [11,12,13,14,15].

Particularly in fatigue loading, many different factors influence the damaging process, e.g., the type of fatigue load (mean load, load ratio, testing frequency, load sequence, etc.), environmental conditions (temperature, humidity, etc.), material configuration and manufacturing processes [16,17]. Furthermore, load reversals are a common issue and their detrimental effect on damage accumulation is getting increasing attention [18,19,20,21,22].

Composite microcracking is in focus of research for many years. Early works from Reifsnider et al. [23,24,25] as well as Crossman et al. [26,27] analysed the complex damage process within composites and pointed out the main damage mechanisms microcracking, delamination and fibre failure as well as their interaction. They linked the damage mechanisms and their effect on the laminate properties, enabling them to derive analytical and numerical models to describe the observed microcracking phenomena and their impact on stiffness loss. The corresponding experiments where conducted in terms of quasi-static or tension-tension fatigue loading only and do not address load reversals. Furthermore, O’Brien [28,29] considered the initiation and growth of delaminations growing from the specimen edges as well as from microcracks under quasi-static tension loading. It was found, that for thin $90^{\circ }$-layers in a cross-ply laminate, delamination initiation is always preceded by microcrack formation. However, when the thickness of the $90^{\circ }$-layer is large (high number of neighbouring $90^{\circ }$-plies) microcracks and delaminations initiate at the same strain level. O’Brien further derived an analytical formulation to predict the strain level and corresponding strain energy release rate for delamination initiation in cross-ply laminates with arbitrary ply-thickness. However, the proposed model is insensitive to the distance between neighbouring microcracks, thus neglecting the effect of crack shielding.

Gamstedt et al. [18] discussed the active mechanisms in fatigue crack formation under tension-tension and tension-compression loading from a micromechanical perspective. They emphasise that a fibre matrix debond induced in tension loading can grow under compression loading due to shear stresses and the remaining crack tip opening at the fibre matrix interface. The opening of the crack tip under compression loading was experimentally observed by microscopic investigations on a model specimen consisting of a transversally loaded single fibre.

Quaresimin et al. [22] used glass fibre reinforced-epoxy (GFRP) tubes to experimentally analyse the influence of load ratio and load biaxiality on the initiation and growth of microcracks. They were able to show, that increasing load ratios lead to a delayed crack initiation as well as slower cyclic crack growth.

Similar findings for carbon fibre reinforced (CFRP) cross-ply laminates tested at different load ratios published by the authors in earlier work [21] support these findings. The results of the fatigue tests on CFRP strip specimens show a more pronounced crack growth for load ratios R<0, compared to experiments with R≥0. By use of an extended damage model, which incorporates the energy release rate (ERR) portion of the compression half cycle into the energy calculations, it is demonstrated that the cyclic crack growth laws for different load ratios collapse into one single scatter band.

The microcracks within the embedded off-axis layer are often origins for the initiation and growth of inter-layer delaminations. Delaminations can grow fast with catastrophic effect on laminate stiffness, strength and stability, because there are no crack stopping mechanisms between the two adjacent layers. Reviews by Bak et al. [16], Khan et al. [30] and Tabiei and Zhang [31] give an extensive overview on the factors affecting delamination initation and growth and summarise a variety of existing models. It is presented, that the ERR threshold for delamination growth $G_{th}$ is independent of the mode-mixity, but strongly depends on the load ratio. Further, numerous influences on fatigue delamination growth, e.g., temperature, matrix brittleness or maximum load, are discussed.

Kardomateas and Malik [19,20] analysed delamination growth and post-buckling behaviour in GFRP and CFRP laminates under cyclic compression loading. They pointed out that the stress state at the delamination crack tip has a mixed mode character with a high amount of mode II and the fatigue growth behaviour strongly depends on the relative through-thickness location in the laminate. Furthermore, it was shown, that a delamination of the same geometric size under identical loading conditions is growing faster in CFRP than in GFRP specimens, hence delamination growth also strongly depends on the material configuration. However, this does not hold for the mode mixity, which was found to be nearly equal in both cases.

This short literature review indicates that most research focusses on one damage mechanism or loading in tension or compression seperately. There is still a lack of experimental evidence and explanations of the detrimental effect of load reversals on the fatigue damage behaviour of composite laminates. Therefore, the aim of the presented study is to provide experimental results, further insight into the mechanisms which govern the fatigue failure of composite laminates and to derive explanations for the observed phenomena with special attention to the influence of tension-compression loading. Further attention has to be paid to residual stresses affecting composite materials due to their highly anisotropic material properties, especially in case of CFRP cross-ply laminates [21,32]. The correct description of the failure inducing stresses within a layer therefore requires the determination of these residual stresses within each layer. It is of further interest to know if the microcracks close under load reversal and readmit load carrying capabilities of the $90^{\circ }$-layer. Hence, a detailed analysis of the crack opening with and without loading is performed alongside the determination of the crack density, the crack angle of individual microcracks and the initiation and growth of delaminations under cyclic loading.

## 2. Materials and Methods

### 2.1. Specimen Preparation

Different specimen types were manufactured to determine the elastic and strength properties of the single layer as well as failure behaviour of cross-ply laminates. The single layers consist of T700SC 6k carbon fibre rovings from Toray International Inc. and Araldite LY556 epoxy resin system from Huntsman LLC.

In a first step, the unidirectional (UD) as well as the bidirectional (BD) lay-up is built by a dry winding process on to a plate-type winding core with rounded edges providing two laminate stacks at a time with a layup of $\left[0_{11}\right]$ and $\left[0_{2}/90_{7}/0_{2}\right]$ for unidirectional and cross-ply laminates, respectively. In case of the cross-ply laminates, the winding core is manually rotated about $90^{\circ }$ twice during the winding process to realise both fibre directions. The dry preform, which is still attached to the winding core, is then infiltrated with the resin system by means of resin transfer moulding (RTM). The UD laminates were infiltrated along the fibre direction and the BD laminates were infiltrated along the fibre direction of the $0^{\circ }$-layers. To avoid dry areas or voids within the laminate a high pressure of $p_{i}=7.5$ bar is applied and held for about one hour. As the curing process is started, the pressure is lowered to $p_{c}=2.5$ bar to avoid bulging of the mould in order to produce laminates with low thickness variation. The laminates are cured for 3 h at 80 ${}^{\circ }$C inside the mould, afterwards demolded and cut down from the winding core and finally post-cured at 150 ${}^{\circ }$C for 4 h in a convection oven. The fibre volume fraction of the laminates has been determined to $\varphi_{f}=60.87\pm 0.84$%. Fibre misalignment has been checked by visual inspection at the top and bottom layer of the laminates and has been found to be negligable. However, fibre misalignment within the central $90^{\circ }$-layer was not checked.

**Figure 1:**
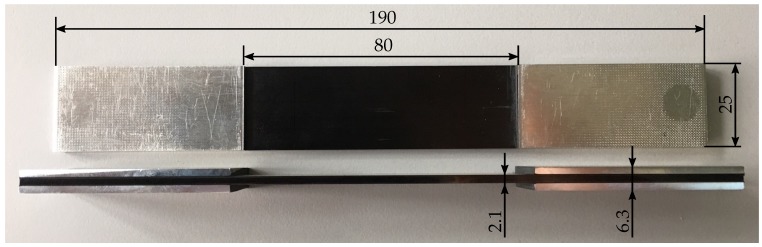
Image of a representative cross-ply specimen used in the present study for static and fatigue tests and the corresponding dimensions in millimetres (mm).

The specimens used for material characterisation were cut by water-jet to their specific dimensions according to the corresponding standards [33,34,35,36,37]. The cross-ply specimens used in the fatigue experiments and the microcracking analyses were cut by a high precision abrasive cutting machine with a feed of $v_{feed}=0.05$ mm/s to avoid initial flaws at the specimen edges. Aluminium end tabs with a thickness of 2 mm and 55 mm length are applied to the cross-ply composite plates using the epoxy based glue SCOTCH WELD DP-490 by 3M. To alleviate buckling tendency of the cross-ply specimens under compression loads the free length used in fatigue testing is reduced to 80 mm and the specimens have a total length of L=190 mm, width W=25 mm and a laminate thickness of B≈2.1 mm. The nominal ply thickness of the single layers of the specimens is t=0.182 mm. Finally, the specimen edges of the cross-ply specimens are polished up to a grain size of 5 μm to minimize edge effects and ensure perceptibility of the microcracks. An image of a representative specimen is given in Figure 1.

### 2.2. Material Characterisation

The elastic properties of the composite material are determined by testing of unidirectional specimens in terms of tensile, compression and shear (V-notched rail shear, VNRS) experiments. Strain gauge rosettes are applied to both sides of the specimens loaded in tension and compression to monitor possible bending and to determine the Poisson’s ratios. Most of the specimens exhibit bending strains below 5%. If the tested specimens show higher bending strains throughout the test, the testing results are rejected and not considered in the calculation of the mean and standard deviation of the material properties. The VNRS experiments are accompanied by digital image correlation (DIC) for strain measurement. Therefore it is possible to observe the strain field within the complete notched area. Subsequently, the shear modulus is calculated from the stresses and strains between the two notches. The elastic material properties are given in Table 1.

It should be noted, that the standard deviations of the fibre parallel compressive strength $R_{∥}^{c}$ as well as the corresponding elastic modulus $E_{∥}^{c}$ are considerably high. To account for thermal stresses the coefficients of thermal expansion (CTE) are determined by dilatometer measurements of 20 specimens with a heating rate of 1 K/min parallel and perpendicular to the fibre direction, respectively. A comparable curing state between the different composite plates is assured by determining the glass transition temperature $T_{g}$ in terms of differential scanning calorimetry (DSC) measurements from three specimens taken randomly from laminated composite plates. The mean values and standard deviations of the strength properties, failure strains as well as the thermal properties are given in Table 1.

### 2.3. Quasi-Static Testing Procedure

The quasi-static analysis of the cross-ply laminates is performed by means of a Zwick Z250 testing machine with a 250 kN load cell at standard atmosphere (23 ${}^{\circ }$C and 50% r. h.) with a cross-head speed of v=2 mm/min. The specimens are tested incrementally in axial tension to determine the onset and propagation of microcracks with increased loading. After each loading step the specimens are removed from the testing machine and examined by microscopic imaging to determine the edge crack density. By normalising the averaged number of cracks on both specimen edges with the observation length $L_{0}$, the crack density $c^{i}$ is determined after each loading step *i* according to

(1) $$c^{i}=\frac{n_{cracks,1}^{i}+n_{cracks,2}^{i}}{2L_{0}},$$

where $L_{0}$ is 50 mm. Two specimens are tested with different load increments (1 kN and 2 kN) to check for significant influence of the load increment and number of specimen removals from the testing machine on the crack density evolution.

To exactly determine the onset of microcracking, the experiments are accompanied by acoustic emission (AE) performed by the help of a Vallen Systeme AMSY-5 measurement system with two wideband sensors of the type VS150-M with a frequency range from 100 kHz to 450 kHz. The sensors were attached to the top and bottom at the same side of the specimen. Table 2 summarises the acquisition parameters used for the measurements. The cumulated acoustic energy is used as a parameter to detect the onset of microcracking, because minor damage mechanisms as interface fracture or environmental effects generate only small amounts of acoustic energy, hence the onset of inter fibre failue is clearly dinstinguishable. Furthermore, fibre breaks generate large amounts of acoustic energy and can therefore be seperated as well.

### 2.4. Fatigue Testing Procedure

**Figure 2:**
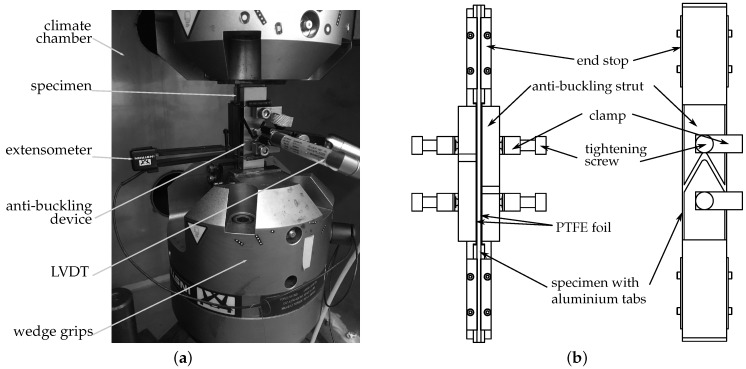
(**a**) Photograph of the test setup used for cyclic testing with load reversals and (**b**) schematic illustration (top and side view) of the assembled anti-buckling device showing the individual components.

The cyclic experiments are conducted on a servo-hydraulic Instron 8801 universal testing machine equipped with a 100 kN load cell and mechanical wedge grips. All tests are performed at constant stress amplitudes and load control. To avoid buckling and premature failure due to geometrical stability issues, the specimens are supported by an anti-buckling device clamped onto the specimen. Polytetrafluorethylen (PTFE) films are placed between the contact areas of the specimen and the anti-buckling device to minimise friction and avoid abrasion. The laminate strain is measured by use of an extensometer attached to one of the free specimen edges. A linear variable differential transformer (LVDT) is used to monitor possible bending throughout the cyclic experiments and to stop the test in case of undesired buckling. Additional utilisation of a climate chamber avoids unpreferred specimen heating and reduces the influence of environmental effects. A photograph of the test setup as well as a schematic of the anti-buckling device are given in Figure 2a,b, respectively.

Four different load ratios according to R={0;−1;−3.26;∞} are tested with two specimens, respectively, at a maximum tensile stress of $\sigma_{max}^{lam}=100$ MPa and 105 MPa for tension and reversed tests. A maximum compression stress of $\sigma_{min}^{lam}=-380$ MPa and −400 MPa for pure compression loading is applied. All tests are performed at a testing frequency of 6 Hz. At the beginning and at the end of each fatigue experiment ten characterisation cycles are performed with a testing frequency of 1 Hz and a sampling frequency of 1000 Hz to determine the cyclic hysteresis before and after testing.

The test schedule is summarised in Table 3. Specimens with a maximum stress of $\sigma_{max}^{lam}=105$ MPa and $\sigma_{min}^{lam}=-380$ MPa are tested in an intermittent manner and are removed from the testing machine at distinct numbers of cycles. Micrographs of the polished specimen edges are used to evaluate the crack density, fibre angles and (edge-) delamination lengths after each testing interval. The crack density is also measured in terms of the number of microcracks averaged over both specimen edges according to Equation (1), whereas crack angles and delamination lengths are determined only at one specimen edge. Crack counting for the second set of specimens was performed by edge replica technique with use of a dye penetrant and adhesive tape during the experiment and by optical microscopy after testing. The replica technique does not allow the quantification of delamination growth, hence only the final delamination lengths after $n=10^{6}$ load cycles were measured for this set of specimens. All specimens have been checked for initial flaws at the specimen edges by optical microscopy. Except for specimen no. 4 (see Table 3), no initial flaws or manufacturing defects were found. It should to be mentioned, that reproducibility of the crack counting results should be analysed within future experiments. However, crack angles and delamination lengths were determined from numerous individual cracks and are therefore considered to be representative.

## 3. Results

### 3.1. Incremental Static Tension Loading

**Figure 3:**
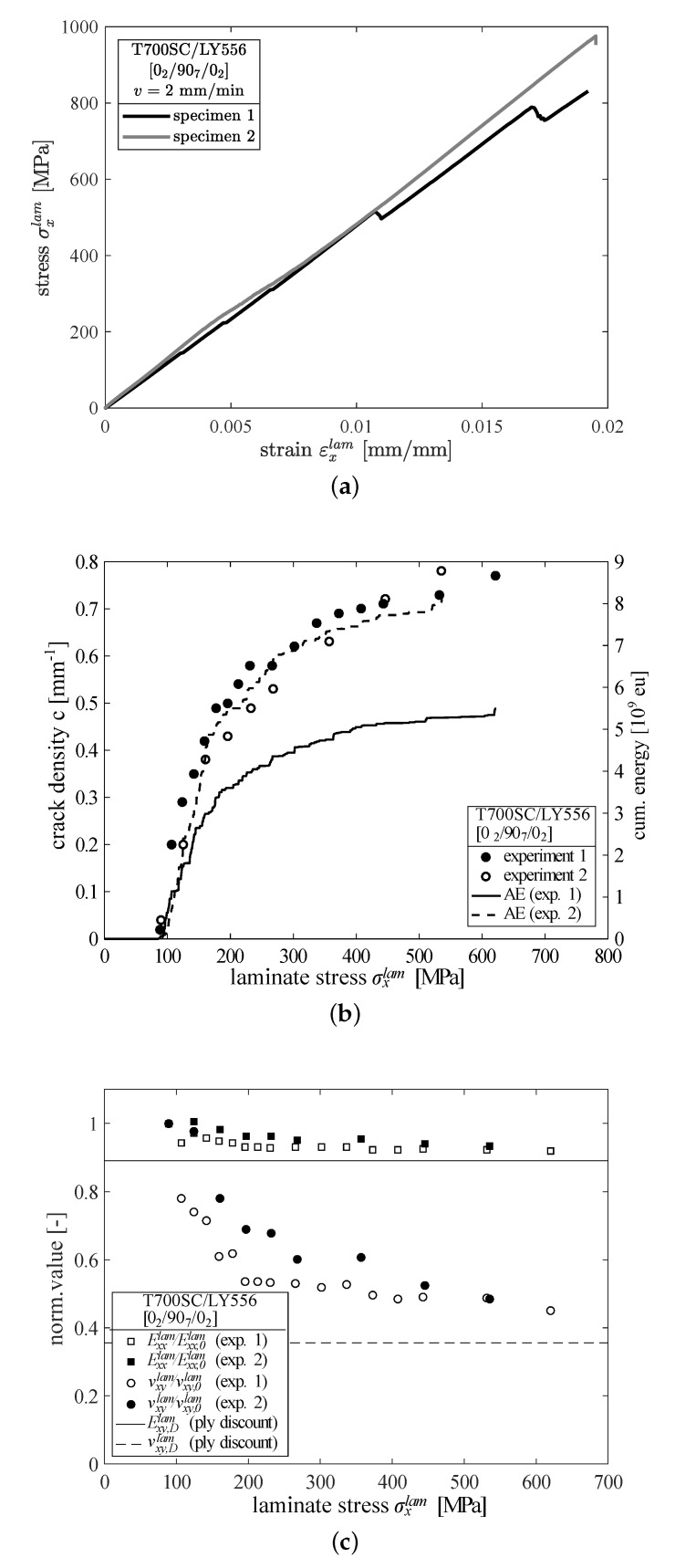
(**a**) Stress-strain curves for two laminate specimens tested until final failure, (**b**) crack density evolution of CFRP cross-ply laminates loaded in axial tension and corresponding cumulated acoustic energy as determined throughout incremental load tests and (**c**) corresponding reduction of axial stiffness and Poisson’s ratio.

The results of the crack density analysis together with the corresponding AE measurements and representative stress-strain curves of cross-ply laminates tested up to ultimate failure are shown in Figure 3. The first cracks that were observed by optical microscopy at a laminate stress of $\sigma_{x}^{lam}=95$ MPa coincide well with the first rapid increase of acoustic energy (cf. Figure 3b). There is a steep increase of the crack density in both specimens up to a laminate stress of $\sigma_{x}^{lam}\approx 250$ MPa. Beyond this point, the crack density as well as the cumulated energy curves are flattening out. This typical behaviour at higher loadings can be explained by the small distances between adjacent cracks and the initiation and growth of delaminations originating from the crack tips. They additionally reduce the length of the undamaged region between the cracks, hence a new crack that forms between two cracks with crack tip delaminations requires higher loadings [38,39]. However, at this point it is not possible to quantitatively evaluate the crack density by means of AE. The shape of the curves reflect the qualitative trends of the crack density, but it is not possible to directly relate the amount of energy to a certain crack density. Furthermore, as seen from Figure 3b, no significant effect of the amount of removals or the choice of the load increment has been found.

In Figure 3c the changes of the laminate stiffness and Poisson’s ratio are shown, normalised by their original undamaged values. Both specimens show a similar maximum stiffness loss of approximately 7% and a maximum reduction of the Poisson’s ratio of 52–55%. Most of the damage takes place from the first initiation of damage at $\sigma_{x}^{lam}=95$ MPa to $\sigma_{x}^{lam}=250$ MPa corresponding well with the steep increase of microcracks. Additionally the axial laminate stiffness and Poisson’s ratio in case of total damaging of the 90${}^{\circ }$-layer (ply discount) are given by the horizontal solid and dashed lines, respectively. The corresponding stiffness loss of approx. 11% and the reduction of the Poisson’s ratio of approx. 64% were calculated by the classical lamination theory (CLT). Furthermore it becomes clear, that subsequent to the formation of microcracks, the initiation and growth of delaminations has negligible influence on stiffness loss, at least in case of tension loading. The effect of delaminations on axial stiffness is expected to be more severe when compression loads are applied to the laminates. From an experimental point of view it is interesting to note, that in contrast to stiffness, the decrease of the Poisson’s ratio is more pronounced, hence it is a more sensitive damage parameter.

### 3.2. Crack Opening Displacement Analysis under Reversed Loading

To further analyse the influence of microcracks on the laminate behaviour it is of interest whether the cracks close under reversed loading or remain open. The crack opening displacement (COD) of a crack is investigated since it has been found to be an important parameter and can be used to calculate the residual laminate stiffness as well as the energy release rate for microcracking [40,41,42,43,44].

**Figure 4:**
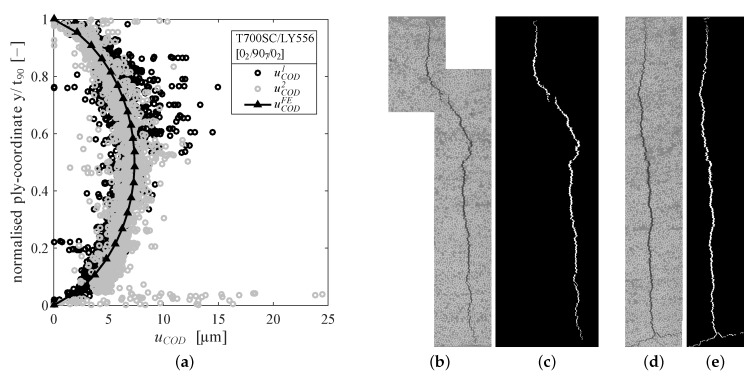
(**a**) Crack opening displacements measured by optical microscopy and calculated from FE-simulation with residual stresses and $\Delta T=T_{room}-T_{cure}=-110$ K and corresponding microscopic images of inter fibre cracks 1 (**b**,**c**) and 2 (**d**,**e**), respectively. The averaged CODs are ${\tilde{u}}_{COD}^{1}=$ 5.34 μm and ${\tilde{u}}_{COD}^{2}=$ 5.85 μm [21].

As pointed out in the literature the amount of residual stresses in a laminate is important to be considered in an appropriate damage analysis [32,45]. In a first step a cross-ply specimen was cyclically loaded for n=5000 cycles under fully reversed (R=−1) loading with a maximum cyclic stress of $\sigma_{x,max}^{lam}=95$ MPa to introduce microcracks. Only two microcracks, which were far apart from each other, were found at the polished specimen edges. Both cracks were examined by optical light microscopy with 500-fold magnification. The horizontal crack opening was measured by converting the image into a black-white image by thresholding the corresponding grayscale image in MATLAB™. The only remaining white pixels belong to the observed cracks and are summed up row-wise to give the COD. Averaged CODs ${\tilde{u}}_{COD}^{1,2}$ have been determined to ${\tilde{u}}_{COD}^{1}=$ 5.34 μm and ${\tilde{u}}_{COD}^{2}=$ 5.85 μm. The thresholding procedure and the result of the COD measurement are given in Figure 4.

A two-dimensional finite element (FE)-model was set-up in Abaqus CAE to determine the amount of residual stresses leading to the measured CODs. The FE-model consists of fully integrated plane-strain elements (CPE4), periodic boundary conditions at the specimen edges in loading direction and assumes linear elastic material behaviour without delaminations at the crack tips. The elastic properties from Table 1 were used for the model calculations and an initial state corresponding to the curing temperature $T_{cure}=150$${}^{\circ }$C was applied to the undamaged model. The residual stresses after cooling were then applied as initial stress state to the model comprising a crack within the 90${}^{\circ }$-layer. The model was allowed to relax (no constraint in the 0${}^{\circ }$-direction, except for periodicity), leading to separation of the crack faces due to residual stresses. The COD was determined by calculating the difference of the normal displacements at the crack faces within the 90${}^{\circ }$-layer. The corresponding curing temperature that invokes the same COD as measured by light microscopy was found to be $T_{cure}=133$${}^{\circ }$C, yielding a temperature difference of $\Delta T=T_{room}-T_{cure}=-110$ K and residual stresses within the 90${}^{\circ }$-layer of $\sigma_{90}^{res}={\left[-15.9\;25.7\;0.0\right]}^{T}$ MPa. The COD results of the FE-simulation are also shown in Figure 4a. In addition, Brod et al. [46] have performed FE-simulations giving very similar results for the residual stresses in the 90${}^{\circ }$-layer with $\sigma_{90}^{res}={\left[-16.1\;25.1\;0.0\right]}^{T}$ MPa. It is interesting to note, that the temperature invoking the same CODs within the simulation as observed experimentally is well below the curing temperature used in manufacturing. Possible reasons for this mismatch might be the temperature dependence of the CTEs and possible chemical shrinkage of the resin during the curing process, that is not captured by the CTEs. Furthermore, creep and relaxation processes may take place due to elevated temperatures throughout the cooling procedure.

**Figure 5:**
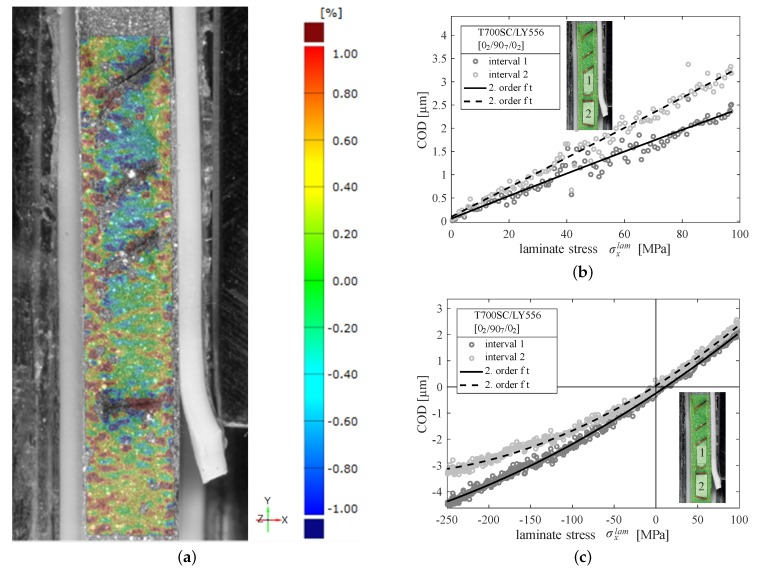
(**a**) Strain field ($\varepsilon_{yy}$) at the specimen edge in presence of four microcracks at $\sigma_{max}^{lam}=322$ MPa as determined by DIC. Loading direction is the *y*-direction according to the coordinate system. Calculated crack opening displacements for a CFRP cross-ply laminate for (**b**) tension and (**c**) tension-compression loading, respectively. During unloading two additional cracks formed as seen in the small pictures. Local strains and CODs are shown for two crack intervals indicated in the small pictures.

Considering load reversals within fatigue tests it is of particular interest to investigate the opening and closing of cracks. Therefore one specimen was studied in detail throughout one load cycle by DIC at the specimen edge and the strains in the cross-ply laminate were examined layerwise (cf. Figure 5a).

First, the specimen has been statically loaded in tension to introduce a significant amount of cracks and subsequently loaded in tension and compression of different magnitudes. Additional to the DIC measurements, the global laminate strains were recorded by an extensometer at the opposing specimen edge. It was therefore possible to analyse the strains between two adjacent cracks experimentally. As seen from Figure 5a, the strains within the $90^{\circ }$-layer are significantly reduced between two adjacent cracks and the 0${}^{\circ }$-layers experience higher strains. In undamaged areas (at the bottom of Figure 5a) the layers exhibit approx. the same strains. However, due to crack formation the applied grayscale pattern is destroyed locally at the crack formation loci.

Assuming linear elastic material behaviour and perfect bonding between the 0${}^{\circ }$- and 90${}^{\circ }$-plies in a cross-ply laminate the COD can be expressed as the strain mismatch between the laminate and the 90${}^{\circ }$-ply within a cracking interval of length 2L leading to the equation [7]

(2) $${\tilde{u}}_{COD}=2\Delta \varepsilon_{x}L=2\left(\varepsilon_{x}^{lam}-\varepsilon_{x}^{90}\right)L.$$

The calculated CODs along the unloading path for pure tension and a tension-compression cycle are given in Figure 5b,c, respectively. It has to be kept in mind, that CODs due to residual stresses were not captured by the experimental procedure. This is due to the fact that the reference condition for the DIC measurements is the undamaged specimen with inherent residual stresses.

Regarding Figure 5b, a linear dependence of the COD on the applied load is oberved, which is in agreement with findings from other researchers [42]. This further confirms, that the cracks remain open when the specimen is completely unloaded due to residual stresses. When loading is continued into compression the relation between the applied laminate stress and the (negative) CODs becomes non-linear indicating crack closing effects (cf. Figure 5c).

Considering an averaged COD of 5.34 μm to 5.85 μm it becomes clear, that even at the maximum applied stress level of $\sigma_{x}^{lam}=-250$ MPa, the cracks do not close completely. With respect to the fatigue experiments it can be stated here, that for a significant amount of nominal compression loading the embedded $90^{\circ }$-layer is still loaded in tension.

### 3.3. Evolution of Microcracks in Fatigue

**Figure 6:**
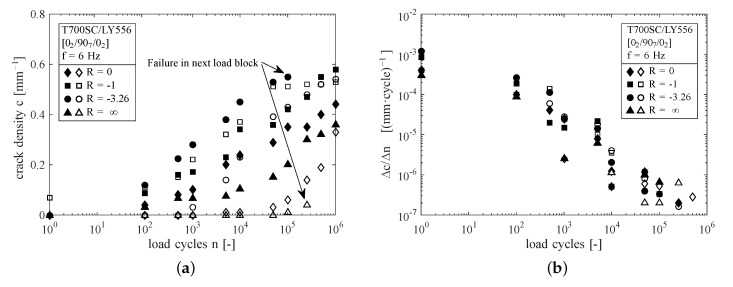
(**a**) Evolution of inter fibre crack density for different load ratios and (**b**) corresponding crack density growth rates versus number of cycles, with a maximum stress $\sigma_{x,max}^{lam}=105$ MPa (filled symbols) and $\sigma_{x,max}^{lam}=100$ MPa (open symbols) for R={0,−1,−3.26} and minimum stress $\sigma_{x,min}^{lam}=-380$ MPa (▴-symbols) and $\sigma_{x,min}^{lam}=-400$ MPa (Δ-symbols) for R=∞, respectively.

The analysis of the fatigue behaviour focusses on the determination of the active damage mechanisms. Hence, the crack development has been observed regarding the aspects: amount of cracks, crack growth rates and cracking angle inclined to the loading direction. The results of the crack counting are given in Figure 6a for all tested specimens. Except for one specimen (□-symbols), all specimens were free of microcracks at the beginning of the test. The pre-damaged specimen had six cracks at one specimen edge, not spanning the entire specimen width. They were therefore considered to be short edge cracks as a consequence of the residual stresses after manufacturing.

In general the microcracking process is more pronounced in case of reversed loading (R<0), which has also been found by [47]. Considerably lower cracking is observed at lower stress levels for R=−3.26 and R=0. However, in case of R=−1 both specimens show comparable amounts of cracks. Crack initiation is also shifted to higher amounts of load cycles for lower load levels, except for the initially cracked specimen. Surprisingly, both compression loaded specimens show a significant amount of perpendicular cracks. Furthermore, the specimen stressed with $\sigma_{x,min}^{lam}=-400$ MPa (Δ-symbols) shows less microcracks compared to the specimen loaded with $\sigma_{x,min}^{lam}=-380$ MPa (▴-symbols). Only two of the specimens (indicated in Figure 6a) failed throughout the tests by local buckling in compression loading.

The crack density evolution per load cycle versus the applied loading cycles is given in Figure 6b. The rate of crack density evolution decreases during the experiments, which is a direct consequence of the cracking process itself, because of increasing crack density. Due to the neighbouring cracks, crack interaction leads to a reduction of the remaining stresses between two adjacent cracks and therefore delays the cracking process. This is also termed crack shielding in the literature. However, no significant differences in terms of crack density evolution rate can be found between the load ratios.

**Figure 7:**
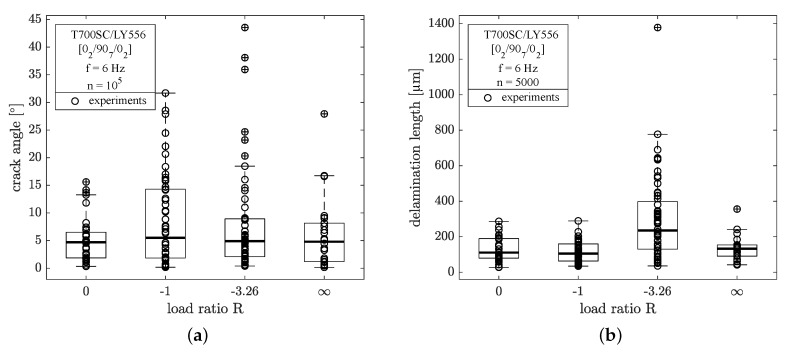
(**a**) Boxplot of crack angles after $n=10^{5}$ and (**b**) length of delaminations initiated after n=5000 load cycles for different load ratios (outliers are marked by ⊕-symbols).

From edge investigation by light microscopy the crack angles of the microcracks have been recorded for one set of the specimens after $n=10^{5}$ cycles. The crack angle was measured versus the normal of the load direction vector, hence a crack angle of $0^{\circ }$ denotes a perfectly perpendicular crack with respect to the loading direction. In all cases the crack angle has been determined for the upper and lower half of the $90^{\circ }$-ply to account for non-straight crack shapes (e.g., triangle or crooked shapes). In Figure 7a, it is shown, that the median values of crack angles for all load ratios do not differ significantly from another at approx. $5^{\circ }$. As stated before, the crack formation for the specimen loaded in pulsating compression is also mainly perpendicular to the loading direction. In case of load reversal (R={−1,−3.26}) much higher scatter of data is encountered with higher crack angles compared to pulsating loads.

### 3.4. Delamination Growth during Cyclic Loading

The initiation and evolution of interlaminar delaminations has been recorded in addition to the analysis of microcrack formation. Delaminations were measured from the same pictures used for the determination of the cracking angle of the microcracks. The top and bottom delaminations at the $0^{\circ }/90^{\circ }$-interfaces starting from a microcrack were quantified from tip to tip for every crack that initiated within the first n=5000 cycles. Delaminations at the tips of subsequently initiated microcracks were not considered in the analysis.

In Figure 7b the delamination lengths after n=5000 cycles are given. It becomes clear that the loading in tension-compression with a high amount of compressive stresses (R=−3.26) leads to a more severe formation of delaminations in the early stage of the fatigue experiment. For all other load ratios the length of the delaminations are comparable at approx. 150 μm, with much lower scatter. Due to the differences in the microcracking process, the amount of cracks considered for this investigation varies between the load ratios. Some delaminations were bigger than the microscopic image region, so their length could not be determined, but the number of very large delaminations has been seperately counted for each load ratio. An overview of the analysed data for delamination growth is given in Table 4, where ${\bar{a}}^{ini}$ denotes the average initial delamination length, ${\bar{a}}^{10^{5}}$ the length after $n=10^{5}$ load cycles, ${\bar{a}}^{10^{6}}$ is the delamination length at the end of the experiment ($n=10^{6}$) and No.${}^{large}$ represents the number of delaminations larger than the image region. These delaminations were not considered in the determination of ${\bar{a}}^{10^{5}}$ and ${\bar{a}}^{10^{6}}$. Hence, the total delaminated length at the end of the test is bigger than ${\bar{a}}^{10^{6}}$ when No.${}^{large}>0$. The total amount of the considered delaminations (data sets) depends on the number of initiated microcracks and therefore varies widely between the load ratios.

**Figure 8:**
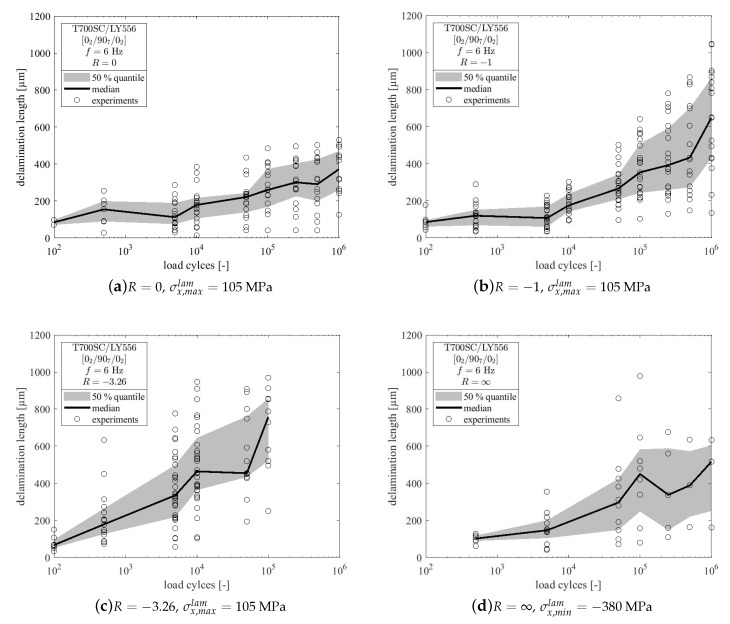
Evolution of edge delamination length as observed at one edge for (**a**) *R* = 0, (**b**) *R* = −1, (**c**) *R* = −3.26 and (**d**) *R* = ∞. Delaminations at the top and bottom of the 90°-ply are treated separately. Delaminations larger than the image region are not considered within the calculation of median and 50%-quantile values.

After n=100 load cycles the experiments were stopped for the first time. At this time all specimens with a high amount of tensile stresses showed minor delaminations at the crack tips and for R=0 the lowest amount of delamination was found. In case of pulsating compression load the first delaminations were observed after n=500 load cycles with a mean delamination length of ${\bar{a}}_{\infty }^{ini}=101.1\;\pm \;$ 23.8 μm. From Table 4 it becomes clear, that the largest initial delaminations were found in case of reversed loading with high amount of compressive stresses (R=−3.26). Throughout the experiment, delamination lengths increase in all cases and the specimens loaded with compressive stresses show larger delaminations than the purely tension loaded specimen. The detailed evolution of delamination growth is depicted in Figure 8 for all load ratios. In all cases data scatter is high, so median values and 50%-quantiles of the recorded data are additionally shown in Figure 8.

Two specimens (no. 5 and 8 from Table 3) failed during the fatigue tests by local buckling within the compression load cycle. These two specimens were stressed up to $\sigma_{x,min}^{lam}=-342.3$ MPa and $\sigma_{x,min}^{lam}=-400$ MPa, respectively.

Furthermore, the fracture surfaces of the delaminated areas have been analysed by scanning electron microscopy (SEM). Therefore, the remaining part of the $0^{\circ }$-layer was mechanically removed and the fracture surface was analysed on the $90^{\circ }$-ply.

**Figure 9:**
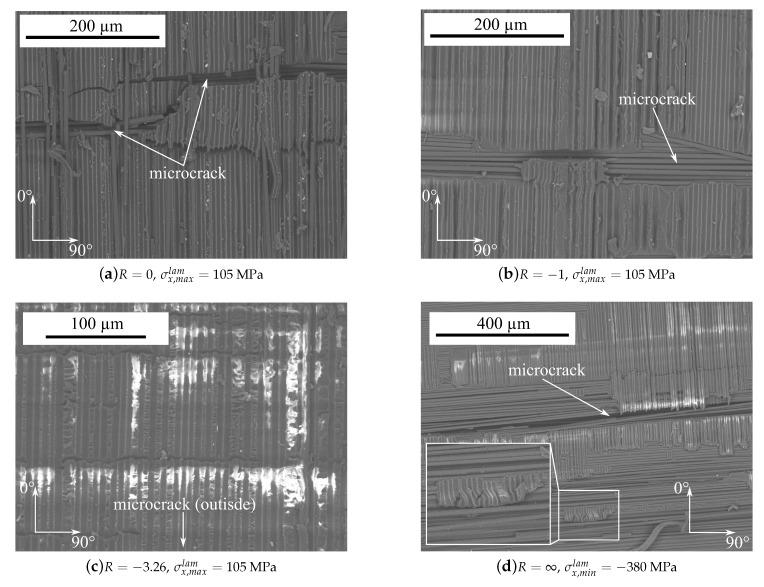
SEM images showing fracture surfaces of crack tip delaminations for different load ratios after *n* = 10^6^ load cycles (white areas occur due to electric charging of the material). The kink band in (**d**) is magnified.

Regarding the images in Figure 9a,b, for the stress ratios R=0 and R=−1 the fracture surface appears smooth and the fibre imprints within the remaining resin can be seen very well. Shear cusps can barely be seen and a little amount of debris is present around the crack tip. In contrast, the fracture surface for R=−3.26 (Figure 9c) appears rough with shear cusps within the resin rich areas between two adjacent fibres. The fracture surface is also traversed by horizontal linear indentations spanning several hundred micrometers. The microcrack of the adjacent $90^{\circ }$-ply is situated beyond the bottom of the image. Considering Figure 9d showing the fracture surface for R=∞, the amount of remaining resin is, compared to the other fracture surfaces, remarkably low. The fracture surface is mainly smooth and free of shear cusps. In the lower middle part of the image the formation of a kink band can be seen from the remaining fibre imprints. For better perceptibility, the kink band is additionally magnified within Figure 9d.

## 4. Discussion of Results

### 4.1. Influence of Residual Stresses

From the results shown in Section 3.2 it becomes clear, that residual stresses play an important role for the stress state in embedded plies, especially in highly anisotropic materials like CFRP. Concerning the $90^{\circ }$-layer, the transverse residual stresses reach a value of $\sigma_{22,res}^{90}=25.7$ MPa which is equal to 71% of the UD transverse strength and might be the reason for the initial edge cracks in one of the specimens. Small precracks from the cutting and polishing process and the mismatch of the Poisson’s ratio may lead to further stress magnification and promote the formation of initial edge cracks due to residual stresses. Regarding the cyclic experiments, these residual stresses can be interpreted as an additional mean stress changing the load ratio in the layers. For the transverse layer the local load ratio $R_{loc}^{90}$ can be calculated by

(3) $$R_{loc}^{90}=\frac{\left(\sigma_{m}^{90}+\sigma_{res}^{90}\right)-\sigma_{a}^{90}}{\left(\sigma_{m}^{90}+\sigma_{res}^{90}\right)+\sigma_{a}^{90}}\;\mathrm{with}\;\sigma_{m,a}^{90}=\frac{E^{90}}{E_{x}^{lam}}\sigma_{m,a}^{lam},$$

where $\sigma_{m}^{90}$, $\sigma_{a}^{90}$, $\sigma^{lam}$ and $E_{x}^{lam}=50.5$ GPa denote the mean stress, stress amplitude, remote laminate stress and laminate modulus, respectively. From Table 5 it can be seen, that the local load ratio within the $90^{\circ }$-layer is severely influenced by the residual stresses.

Consequently, the macroscopically applied load ratios are shifted into the direction of pulsating tension loading, meaning that the macroscopically fully reversed experiments (R=−1) actually lead to pure cyclic tension loading within the $90^{\circ }$-layer. Furthermore, the lowest stress amplitudes are found for R=0 and the highest for R=−3.26. As seen from Figure 6a most of the specimens show first cracks after the first examination at $n=10^{2}$ cycles, but the crack density obviously evolves differently. This is interesting, because for R={0;−1;−3.26} the maximum ply-stresses are the same, but they exhibit different stress amplitudes. However, this does not hold valid in case of R=∞ where stress amplitudes are high, but crack growth is slow. This might be a consequence of the higher compression loadings, which are applied within every load cycle. In fact compression loading would lead to shear failure of the embedded layer, where, in general, fracture stresses are higher compared to transverse failure in tension. Hence, compression failure appears to be unlikely and transverse failure is initiated due to pulsating residual stresses in case of macroscopically pulsating compression loads. Furthermore, the evaluation of the crack angles in Figure 7a does not reveal a significant increase of the fracture angle for R=∞. It is stated, that microcracks are introduced by tensile stresses, even in case of compression loading. Here, the pulsating residual stresses lead to crack formation and growth. Due to the high amount of compression loading, crack growth is slow and delamination growth from the crack tips is the prevailing damage process.

### 4.2. Influence of Load Reversal on Delamination Growth

The second issue of interest is the initiation and growth of delaminations. The fatigue growth behaviour varies between pulsating and reversed loading, as it can be seen from Figure 8a–d. In case of pulsating tension or compression loading (R=0 and R=∞) delamination growth is rather slow compared to the reversed loading cases. Furthermore the total number of delaminations was lower due to less microcracking in pulsating loading and the average delamination lengths at the end of the tests ($n=10^{6}$) were also lower than in case of reversed loading. Due to the fact that delaminations, which were bigger than the image region of the microscope, were not quantified, Figure 8a–d does not account for the large delaminations listed in Table 4. Hence, to assess the criticality of the applied load ratios, the lengths of minor delaminations at the end of the experiments ${\bar{a}}^{10^{6}}$ as well as the number of delaminations, which are bigger than the image region No.${}^{large}$ from Table 4 have to be considered. Consequently, it can be claimed that high compressive loadings promote delamination growth in the presence of microcracks.

**Figure 10:**
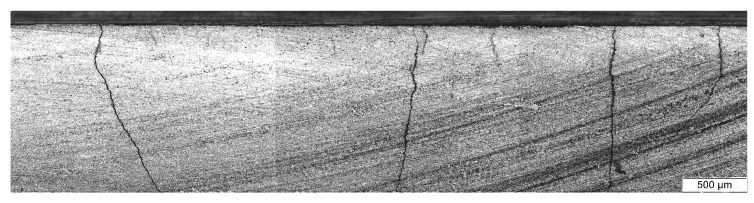
Stitched image of the specimen edge after $n=10^{6}$ cycles for R=∞. The total length of the image is about 5.7 mm. The top $0^{\circ }$-layer is separated along the whole image length and beyond by delamination.

As stated before, two specimens failed prior to $n=10^{6}$ due to local buckling within the compression load cycle. These specimens were tested at stress ratios R=−3.26 and R=∞, implying the highest compression loadings. As seen from Table 4 these specimens had large delaminations, which were often bigger than the image region of the microscope. It can therefore be stated, that delamination growth is much more pronounced in case of load reversals. This results in widespread ply separation and the $0^{\circ }$-layer is not supported by the adjacent transverse layer. Consequently, the $0^{\circ }$-layer takes all the applied load as long as the microcracks do not close. The micrographs further indicate that the delaminations are much larger than the visible image region of the microscope with a length of 2.2 mm. A stitched image, as shown in Figure 10, proofs delamination lengths of 5.7 mm and above. However, local buckling could also be influenced by fibre misalignments or waviness, fibre kinking or local abscence of contact to the anti-buckling device. Thereof only fibre kinking has been observed, as seen in Figure 9d. Additionally, the fibre misalignemts are expected to be small, due to the winding process and the applied pretension of the fibres.

A reason for the differences in delamination growth between specimens tested with and without load reversals might be the change of fracture modes that are active throughout the cyclic loading. The delamination in tension loading can mainly grow under mode II, whereas delamination growth under compression loading has a significant mode I contribution at the tip of the delamination as observed by [20]. This might favor delamination growth under compression loads, wich is further amplified by the through-thickness stresses as a consequence of the microcracks. It is also known, that the mode I fracture toughness is in general much smaller than mode II fracture toughness [48,49], so delamination growth under compression loading might be more pronounced. It should be noted that the specimens no. 6 (R=−3.26) and 7 (R=∞), which did not fail up to $n=10^{6}$ cycles also show extremely widespread delaminations, whereas this is not the case for R=0 and R=−1.

In contrast to these findings based on residual crack opening, Wevers et al. [50] proposed that microcracks in cross-ply laminates already close for load ratios of R≤0.5 due to debris at the crack surface. These results are based on AE measurements. Although the presence of debris at the crack surface can not be excluded here, the results in Figure 5b clearly indicate, that the cracks remain open even for lower load ratios, because the slope of the curve does not change until a laminate stress of $\sigma_{x}^{lam}=0$ MPa. Furthermore, Wevers et al. did not consider residual stresses and the observed pronounced delamination growth for R=0.03 compared to R=0.5 may also be explained by the higher stress amplitudes that were applied to this specimen.

It should be pointed out, that the results obtained here are acquired from a comparatively small set of eight specimens. The crack density evolution in Figure 6a could therefore be affected by scatter, due to manufacturing induced flaws or subsurface defects. However, each specimen comprises many cracks and crack tip delaminations which have been analysed individually, thus leading to profound results in terms of crack angles and delamination growth. To ensure reproducibility and significance, larger specimen sets are analysed in future work.

## 5. Conclusions

The study presents experimental results for the microcracking process in CFRP-laminates in static and cyclic loading. It further provides data for the fatigue growth of inter-layer delaminations emanating from the tips of inter fibre microcracks and the remaining crack opening displacement due to manufacturing induced residual stresses in a cross-ply laminate.

The results show the significant influence of residual stresses to fatigue loading conditions by shifting the local load ratios within the $90^{\circ }$-layer towards pulsating tension loading. No significant difference of the crack angle between the load ratios was found and it is therefore argued, that the cracks initiate due to tension loading. Therefore, the formation of cracks in compression loading was explained by pulsating residual stresses.

By use of microscopic imaging, the residual crack opening displacement was measured and used to calculate the residual stresses within the $90^{\circ }$-layer by means of FEM. It could further be shown by DIC measurements, that the microcracks remain open, even in case of compression loading, hence influencing the local load ratio and compelling local load redistribution into the $0^{\circ }$-layers up to a certain amount of compression loading.

From edge investigations the delamination growth was measured for each load ratio. It was found, that delamination growth is more pronounced in case of reversed loadings (R<0) with a high amount of compressive loading. Delaminations grew from the crack tips up to a few hundred micrometers (R={0,−1}) or even several millimeters (R={−3.26,∞}). In case of high compression loads, the large delaminations lead to final failure of the specimens due to local buckling. It is argued by the authors, that the pronounced delamination growth is a consequence of the change of the fracture modes which are active at the delamination front within tension and compression cycles. In case of tension loading, the fracture process at the delamination front is dominated by mode II crack growth, typically having a high fracture toughness. For compression loads the delamination might buckle locally resulting in a mixed-mode fracture process, due to rising mode I components. Mode I fracture toughness is comparably smaller than mode II, hence, delamination growth might be more pronounced in tension-compression due to mode I crack opening. To further clarify the influence of compression loads on fatigue delamination growth additional investigations have to be carried out focussing on the stress-strain state at the delamination crack front and the active fracture modes with respect to microscopic aspects.

## Figures and Tables

**Table 1:** Elastic, strength and thermal properties of T700SC/LY556 composite material.

$E_{||}^{t}$	$E_{||}^{c}$	$E_{⊥}^{t}$	$E_{⊥}^{c}$	$G_{||⊥}$	$G_{⊥⊥}^{*}$	$\nu_{||⊥}$	$\nu_{⊥⊥}^{**}$
[GPa]	[GPa]	[GPa]	[GPa]	[GPa]	[GPa]	[−]	[−]
129.4	110.7	8.05	8.87	3.91	2.85	0.317	0.41
±5.4	±21.7	±0.44	±0.20	±0.19	±0.15	±0.0076	−
$R_{||}^{t}$	$R_{||}^{c}$	$R_{⊥}^{t}$	$R_{⊥}^{c}$	$R_{||⊥}$	$\alpha_{||}^{***}$	$\alpha_{⊥}^{***}$	$T_{g}$
[MPa]	[MPa]	[MPa]	[MPa]	[MPa]	[$10^{-6}/$K]	[$10^{-6}/$K]	[${}^{\circ }$C]
2089	1032	36.2	164.4	52.2	0.5	38	146.4
±53	±221	±5.3	±7.9	±5.4	−	−	±2.4

${}^{t}$—tension, ${}^{c}$—compression, ${}^{*}$—calculated, ${}^{**}$—assumed, ${}^{***}$—at room temperature.

**Table 2:** Acquisition settings for the acoustic emission measurements.

Parameter	Value	Units
Sample rate	10	MHz
Threshold	46	dB
Gain	34	dB
Duration Discrimination Time	0.2	ms
Rearm Time	0.4	ms

**Table 3:** Test schedule for fatigue experiments with intended maximum number of load cycles $N=10^{6}$ and testing frequency f=6 Hz.

No.	*R*	$\sigma_{\max}$	$\sigma_{\min}$	$\sigma_{m}$	$\sigma_{a}$
[−]	[−]	[MPa]	[MPa]	[MPa]	[MPa]
1	0	105	0	52.5	52.5
2	0	100	0	50	50
3	−1	105	−105	0	105
4	−1	100	−100	0	100
5	−3.26	105	−342.3	−118.7	223.65
6	−3.26	100	−326	−113	213
7	∞	0	−380	−190	190
8	∞	0	−400	−200	200

**Table 4:** Overview of delamination lengths within fatigue experiments for different load ratios.

*R*	Data Sets	${\bar{a}}^{\mathrm{ini}}$	${\bar{a}}^{10^{5}}$	${\bar{a}}^{10^{6}}$	No.${}^{\mathrm{large}}$
[−]	[−]	[μm]	[ μm]	[ μm]	[−]
0	24	90.3±46.1	268.9±123.7	323.5±127.1	0
−1	18	92.8±60.4	373.2±149.8	620.1±208.5	2
−3.26	38	254.2±190.9	696.3±226.6	*	21
∞	12	101.1±23.8	453.2±282.4	575.2±83	6

${}^{*}$—Specimen failed after n=201,424 load cycles.

**Table 5:** Local stresses and load ratios in the $90^{\circ }$-layer within the CFRP cross-ply laminate for different macroscopic load ratios *R*.

No.	*R*	$\sigma_{\max}^{90}$	$\sigma_{\min}^{90}$	$\sigma_{m}^{90}$	$\sigma_{a}^{90}$	$R_{\mathrm{loc}}^{90}$
[−]	[−]	[MPa]	[MPa]	[MPa]	[MPa]	[−]
1	0	42.4	25.7	34.1	8.3	0.61
2	0	41.6	25.7	33.7	7.9	0.62
3	−1	42.4	8.9	25.6	16.8	0.21
4	−1	41.6	9.8	25.7	15.9	0.24
5	−3.26	42.4	−28.9	6.8	35.6	−0.68
6	−3.26	41.6	−26.3	7.7	33.9	−0.63
7	∞	25.7	−34.9	−4.6	30.3	−1.36
8	∞	25.7	−38.1	−6.2	31.9	−1.48
